# Residence in Proximity of a Coal-Oil-Fired Thermal Power Plant and Risk of Lung and Bladder Cancer in North-Eastern Italy. A Population-Based Study: 1995–2009

**DOI:** 10.3390/ijerph14080860

**Published:** 2017-07-31

**Authors:** Paolo Collarile, Ettore Bidoli, Fabio Barbone, Loris Zanier, Stefania Del Zotto, Simonetta Fuser, Fulvio Stel, Chiara Panato, Irene Gallai, Diego Serraino

**Affiliations:** 1 Unit of Cancer Epidemiology, CRO Aviano National Cancer Institute, 33081 Aviano (PN), Italy; bidolie@cro.it (E.B.); stefania.delzotto@regione.fvg.it (S.D.Z.); chiara.panato@cro.it (C.P.); serrainod@cro.it (D.S.); 2 Institute for Maternal and Child Health, IRCCS Burlo Garofolo, 34137 Trieste, Italy; fabio.barbone@burlo.trieste.it; 3 Department of Medical and Biological Sciences, University of Udine, 33100 Udine, Italy; 4 Epidemiologic Service, Regional Health Directorate of Friuli Venezia Giulia, 33100 Udine, Italy; loris.zanier@regione.fvg.it; 5 Regional Environmental Protection Agency of Friuli Venezia Giulia, 33057 Palmanova (UD), Italy; simonetta.fuser@arpa.fvg.it (S.F.); fulvio.stel@arpa.fvg.it (F.S.); irene.gallai@arpa.fvg.it (I.G.); 6 Friuli Venezia Giulia Cancer Registry, 33081 Aviano (PN), Italy

**Keywords:** air pollution, coal-fired thermal power plant, oil thermal power plant, geocoded, lung cancer, bladder cancer, north-eastern Italy

## Abstract

This study investigated the risk of lung and bladder cancers in people residing in proximity of a coal-oil-fired thermal power plant in an area of north-eastern Italy, covered by a population-based cancer registry. Incidence rate ratios (IRR) by sex, age, and histology were computed according to tertiles of residential exposure to benzene, nitrogen dioxide (NO_2_), particular matter, and sulfur dioxide (SO_2_) among 1076 incident cases of lung and 650 cases of bladder cancers. In men of all ages and in women under 75 years of age, no significant associations were observed. Conversely, in women aged ≥75 years significantly increased risks of lung and bladder cancers were related to high exposure to benzene (IRR for highest vs. lowest tertile: 2.00 for lung cancer and 1.94 for bladder cancer) and NO_2_ (IRR: 1.72 for lung cancer; and 1.94 for bladder cancer). In these women, a 1.71-fold higher risk of lung cancer was also related to a high exposure to SO_2_. Acknowledging the limitations of our study, in particular that we did not have information regarding cigarette smoking habits, the findings of this study indicate that air pollution exposure may have had a role with regard to the risk of lung and bladder cancers limited to women aged ≥75 years. Such increased risk warrants further analytical investigations.

## 1. Introduction

Agents classified as Group 1 lung carcinogens by the International Agency for Research on Cancer (IARC) include personal habits and occupational and environmental exposures. Although in many populations cigarette smoking is the main cause of lung cancer, other recognized risk factors may have a relevant impact under local circumstances. Such factors include exposure to identified physical and chemical agents and their mixtures and occupational and environmental activities. The latter may entail exposures occurred in or in proximity of some industrial facilities, air pollution due to road traffic and home heating [1,2,3,4,5,6]. Sufficient or convincing evidence about causes of bladder cancer is associated with cigarette smoking, radiation, exposure to aromatic amines, arsenic and inorganic arsenic compounds, *Schistosoma haematobium*, and work in occupations such as aluminum production, painting, and rubber production [7,8,9]. However, a positive association between bladder cancer and outdoor air pollution has recently emerged [10]. With specific reference to the carcinogenic effect of combustion of coal, burning coal inside the home for the purpose of heating or cooking produces particulate and gas emissions that are lung carcinogens (IARC Group I). Additional substances derived from coal, also recognized as carcinogens, are: coal-tar pitch, soot, diesel engine exhaust, and related occupations (i.e., coal gasification, coke production, iron and steel founding). However, the extension and impact of the carcinogenic effects of emissions from coal and coal-oil-fired plants is a matter of debate, especially when such effects are compared with other local sources of air pollution and with alternative modes of energy production.

## 2. Materials and Methods

The city of Monfalcone is located in the Friuli Venezia Giulia region, northeastern Italy, and shares with 13 surrounding municipalities a concentration of industries (a power plant, a large shipyard, a paper mill, and other manufacturing industries) and several transport infrastructures such as a port, airport, and highways.

The coal-fired and oil thermal power plant is located near the city center of Monfalcone since 1965; over time, the power plant has undergone several additions and changes. Until 1969, there was only one coal-fired power generator with a 60-m high smokestack. In 1970, another coal-fired power generator was added along with another 90-m high smokestack. In 1984, two oil power generators were added and the smokestacks were replaced by one single 154-m high smokestack. In 1990, to reduce the suspension of coal dust, the coal conveyor belt was depressurized.

Throughout the years, the residents of the 14 municipalities have set up citizens committees for environmental controls over the emissions of the thermal power plant, and have solicited epidemiological monitoring of the potential adverse health effects associated with air pollution. In 2014, to respond to these public concerns, the Friuli Venezia Giulia Region implemented the “Health & Environment Regional Observatory”. Point and diffuse emissions, within the 14 municipalities, are measured by the Regional Environmental Protection Agency (ARPA-FVG). The coal-fired and oil thermal power plant, other industries, port, airport, home heating, and highways in the study area contribute pro rata to the overall atmospheric concentrations of fine particulate matter (PM_10_), benzene (C_6_H_6_), nitrogen dioxide (NO_2_), and sulfur dioxide (SO_2_). The study area was defined according to a deposition model of the specific emissions of NO_2_ of the coal-fired thermal power plant.

Since 1995, the incidence of cancer in the whole population of the Friuli Venezia Giulia region (1,200,000 inhabitants) is being recorded by the population-based cancer registry (CR-FVG) [11]. 

In this study we obtained the structure of the residential population (by sex, age in quinquennia, and calendar year) using the same methodology applied in our previous investigation [12]. In the study area, 96.3% of residential addresses, extracted from regional healthcare population database between 1995 and 2009, were geocoded. For each person we considered the various exposures to air pollutants, in the study area, due changing residence in the period 1995–2009. The computerized structure of CR-FVG and regional healthcare population database allows a full integration of the two databases. We extracted from the population-based CR-FVG all incident cases of lung (i.e., 817 men and 285 women) and bladder cancers (i.e., 505 men and 157 women) diagnosed during 1995–2009 among the population residing in the 14 municipalities. Twenty-six cases of lung cancer (16 men and 10 women) and 12 cases of bladder cancers (eight men and four women) were excluded from the study, due to missing valid addresses recorded in the regional healthcare population database. We disentangled incidence data by age (in quinquennia), sex, calendar year of diagnosis, and histological subtype (for lung carcinoma). We used the International Classification of Diseases [13] to identify lung cancer (C33, C34) and bladder cancer (C67, D09.0, D41.4). We used the International Classification of Disease for Oncology (ICDO-3) [14] for the classification of histologic subtypes of lung cancer: adenocarcinoma (ADK) squamous cell carcinoma (SCC), and other and unspecified morphologies (OLC). Age-standardized incidence rates (ASRs) on 2001 EU standard population were computed for cancer incidence in the study area and for the estimated cancer incidence derived from the pool of 8 cancer registers (1995–2007) [15].

**Figure 1:**
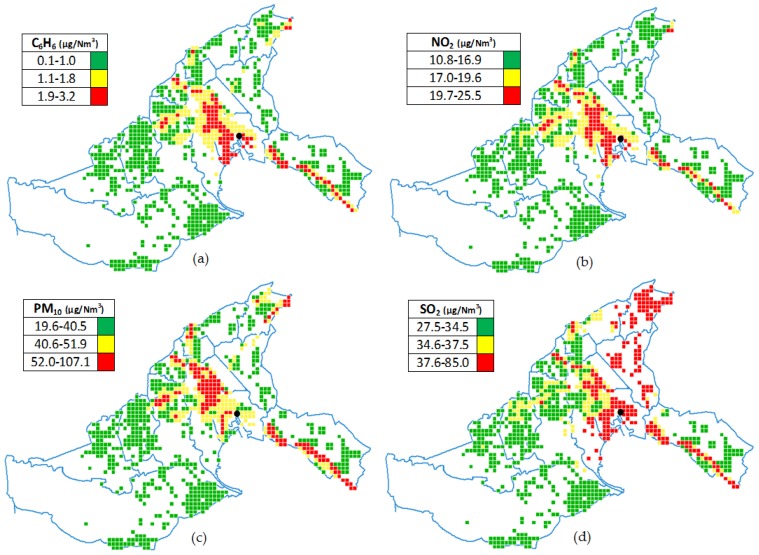
Mathematical modeling of residential exposure to air pollutant in study area, stratified to tertile of exposure: (**a**) Benzene exposure; (**b**) Nitrogen dioxide exposure; (**c**) Particular matter exposure; (**d**) Sulfur dioxide exposure. 400 × 400 m cells correspond to area populated by one or more inhabitants in 1995–2009 period. The blue line corresponds to the boundaries of individual municipalities. The black point corresponds to the location of the thermal power plant.

The residential exposure was defined a priori by ARPA-FVG that recovers the point emission data of C_6_H_6_, NO_2_, PM_10_, SO_2_ reported by individual industries and data on road traffic, port, airport, home heating, and local environmental monitoring system. We considered concentrations of pollutants for which older data were available (1998). Data on pollutants refer to 1998, a year that ARPA-FVG considers as a good proxy of exposure in the study period, taking into account the vehicle fleet, emissions from the power plant, and other industries, and type of home heating. In fact, there is a smooth decrease of anthropogenic emissions over the study area during the past years. The residential exposure was obtained through an integrated approach based on punctual observations and numerical simulated fields [16]. This approach merges punctual monitoring (i.e., precise evaluation of concentration) and the advantages of numerical modeling (i.e., homogeneous fields obtained keeping into account both local meteorological drivers and emission sources). In detail punctual data were obtained through the monitoring network active in the study area. The suite SPRAY (version 3; Arianet Srl, Milan, Italy) is the numerical model applied to this integrated approach [17]. The suite SPRAY is a three dimensional model designed to simulate the airborne pollutant dispersion, able to take into account the spatial and temporal heterogeneity of both the mean flow and turbulence. Concentration fields generated by point, area, or volume sources can be simulated by the model. The trajectory of the airborne pollutant is simulated through virtual particles: the mean motion is defined by the local wind and the dispersion is determined by solving the Langevin stochastic differential equations for the velocity fluctuations, reproducing the statistical characteristics of the turbulent flow. Different portions of the emitted plumes can therefore experience different atmospheric conditions, allowing realistic reproductions of complex phenomena, such as low wind-speed conditions, strong temperature inversions, flow over topography, land use and terrain variability. The merging of the numerical fields and punctual data has been achieved by kriging geospatial technique [18], which is based on prior covariances and can supply the best linear unbiased description of the interpolated values. Then, the residential exposure to air pollution was modelized for the overall area on a 400 × 400 m grid (Figure 1).

Geocoded lung and bladder cancer cases and the population were linked to this grid using Geomedia software, a geographic information system (GIS) (version desktop 2015; Hexagon Geospatial, Stockholm, Sweden). Cases and population were thus exported to compute indicators by a SAS program. An exploratory high exposure (i.e., the highest tertile versus the lowest tertile) analysis was conducted to assess the risk of lung and bladder cancers according to residential exposure to the above mentioned four pollutants. 

Yearly age-standardized (to the 2001 European population) incidence rates (ASRs-EU) per 100,000 inhabitants were calculated for the whole examined period in both sexes and in two age groups (<75 and ≥75 years, i.e., the approximate median age), according to tertiles of exposure. ASRs and their corresponding 95% confidence intervals (CI) were calculated using SAS Enterprise Guide (version 7.1: SAS Institute, Cary, NC, USA). Incidence Rate Ratios (IRRs) and 95% confidence intervals (CI) [19] were computed from the ASRs, considering the lowest tertile of exposure to different pollutants as the reference category.

We calculated annual percent change (APC) [20,21] of incidence rates using Joinpoint software, for the whole 1995–2009 period in both sexes and by the two age groups (<75 and ≥75 years) according to tertile of exposures. Statistical significance (*p* < 0.05) of annual APC was calculated using a Student’s t-distribution [12,20,21].

## 3. Results

Table 1 reports the number of overall incident lung and bladder cancer cases, the ASRs with corresponding 95% CI, according to age and sex. Different incidence rates were observed in lung cancer by histological types in women of all ages with ASR of adenocarcinoma greater than squamous cell ones, and in men aged 75 years or older (i.e., the squamous cell carcinoma -SCC- type was more frequent than the adenocarcinoma -ADK- type).

Table 2 reports the number of lung cancers, the IRRs with corresponding 95% CI, according to age, sex, and tertile of exposure to C_6_H_6_, NO_2_, PM_10_, and SO_2_. No excess risks emerged in both sexes under 75 years of age. An increasing gradient in ASR of lung cancer, according to tertiles of exposure to C_6_H_6_, NO_2_, and SO_2_, emerged only in women aged 75 years or older (Table S1). Only in women aged 75 years or older, the risk of lung cancer increased with increasing degree of residential exposure to C_6_H_6_ (IRR = 1.86, 95%CI: 1.15–3.00 for intermediate tertile vs lowest tertile; IRR = 2.00, 95% CI: 1.23–3.25 for highest tertile), NO_2_ (IRR = 1.70, 95% CI: 1.07–2.71 for intermediate tertile; IRR = 1.72, 95% CI: 1.07–2.77 for highest tertile), and SO_2_ (IRR = 1.55, 95% CI: 0.96–2.49 for intermediate tertile; IRR = 1.71, 95% CI: 1.07–2.73 for highest tertile). The risk of lung cancer with increasing exposure to PM_10_ was not linear: IRR = 2.11, 95% CI: 1.33–3.33 for intermediate tertile, and IRR = 1.57, 95% CI: 0.94–2.60 for highest tertile.

The analysis of the risk of lung cancer was also carried out according to histological subtypes: SCC, ADK, and other lung cancer (OLC) types. A gradient in ASR of lung cancer, according to histological type and tertile of residential exposure to all pollutants, did not emerge in both sexes and in both classes of ages (Table S2). IRRs of lung cancer by histologic subtype (Table S3) highlighted a significantly increased risk of OLC, in women aged 75 years or older, for highest tertile of exposure to C_6_H_6_ (IRR = 2.02, 95% CI: 1.14–3.6), NO_2_ (IRR = 1.70, 95% CI: 0.87–3.00), PM_10_ (IRR = 2.00, 95% CI: 1.11–3.62), and SO_2_ (IRR = 2.54, 95% CI: 1.4–4.63). No excess risk of OLC emerged in women under 75 years of age and in men of all ages. A significantly increased risk of SCC was observed in women aged 75 years or older, for the intermediate tertile of exposure to C_6_H_6_ (IRR = 8.20, 95% CI: 1.06–63.52), NO_2_ (IRR = 8.26, 95% CI: 1.07–63.99), and PM_10_ (IRR = 4.99, 95% CI: 1.13–22.13). This risk was not observed for the highest tertile of exposure to all of the examined pollutants, and no excess risk of SCC emerged in women under 75 years of age and in men in both classes of ages. Moreover, no significant associations with the risk of ADK emerged in both sexes and classes of ages. 

Table 3 reports the number of incident cases of bladder cancer, the IRRs according to age, sex, and tertiles of exposure. An increasing gradient in ASR of bladder cancer, according to tertiles of exposure to all pollutants, emerged only in women of all ages, for example from 9.3 cases/100,000/year with lowest exposure to C_6_H_6_ to 13.4 cases/100,000/year with highest exposure. A similar gradient in ASR of bladder cancer emerged for other pollutants (Table S4). No excess risk emerged in both sexes under 75 years of age, and only in women aged 75 years or older the risk of bladder cancer increased according to increasing degree of exposure to C_6_H_6_ (IRR = 2.39, 95% CI: 1.29–4.44 for intermediate tertile; IRR = 1.94, 95% CI: 1.01–3.74 for highest tertile) and NO_2_ (IRR = 1.97, 95% CI: 1.07–3.63 for intermediate tertile; IRR = 1.94, 95% CI: 1.03–3.65 for highest tertile). The risk of bladder cancer by increasing levels of residential exposure to PM_10_ or SO_2_ was not linear.

The APCs (% per year) of incidence rates of lung cancer for the period 1995–2009 are shown in Table S5. Among men residing in areas with the lowest residential exposure to each pollutant, significantly negative APCs emerged, whereas non-significant APCs emerged among women (Table S5). Men under 75 years of age showed significantly negative APCs in areas with the lowest exposure to all pollutants and in areas with intermediate exposure to C_6_H_6_ (APC = −5.29%; 95% CI: −9.2; −1.2), NO_2_ (APC = −5.50%; 95% CI: −9.6; −1.2), and SO_2_ (APC = 4.8% per year; 95% CI: −7.0; −2.6). Temporal trends showed no variability in APCs among women. The APCs (% per year) incidence rates of bladder cancer for the period 1995–2009 are reported in Table S6. APCs variability was not observed in both sexes and in classes of age or in overall age groups.

## 4. Discussion

According to IARC, air pollution is a Group 1 carcinogen, which causes lung cancer and is associated with an increased risk of bladder cancer [10]. This population-based study assessed the risk of lung and bladder cancers among people residing in the Monfalcone area, northeastern Italy. The area is covered by a population-based cancer registry and it is characterized by the emissions, among other sources, of pollutants from a coal-fired and oil thermal power plant located near the city center.

In men, the incidence rates of lung and bladder cancer for all ages in the study area (Table 1) are lower than the corresponding national incidence rates (82.6 per 100,000 inhabitants for lung cancer; 51.3 per 100,000 inhabitants for bladder cancer). Conversely, in women the local incidence rates are higher than the national incidence rates (18.0 per 100,000 inhabitants for lung cancer; 9.2 per 100,000 inhabitants for bladder cancer).

An excess risk of lung cancer was associated with residential exposure to the highest tertile of C_6_H_6_, NO_2_, PM_10_, and SO_2_ only in women aged 75 years or older—an excess risk restricted to the lung cancer types other than SCC or ADK. Conversely, no excess risk was observed in men of all ages and women under 75 years of age. With regard to bladder cancer, in these women an excess risk was associated with the highest tertile of exposure to C_6_H_6_ and NO_2_ and with an intermediate tertile of exposure to C_6_H_6_, NO_2_, and PM_10_.

The study findings seem to support the hypothesis that the residential exposure to high levels of air pollutants may be associated with an excess risk of incidence of lung and bladder cancers, though the reported excess risks were restricted to older women. The lack of evidence relative to intense exposure to air pollution and lung and/or bladder cancers in men might be explained by heavy smoking habits in both sexes [22] and/or by occupational activities in men (e.g., shipbuilding industries) that put them at high risk of these cancers [7,23,24,25].

The analysis by histological subtypes of lung cancer shows some peculiarities. It is well-known that air pollution in urban areas is a risk factor for ADK [26], while smoking, occupational exposure, and living near pollutant sources may play a more specific role in the etiology of SCC and small cell carcinoma [1,27]. Excess of OLC in women aged 75 years or older living in areas with high exposure to the studied atmospheric pollutants may be due in part to smoking habits, the main cause of small cell carcinoma. It could be also explained by the reduced variability between the lowest and the highest tertile of exposure to all pollutants. Moreover, the association with exposure to C_6_H_6_ or NO_2_ suggests that the excess risk of lung and bladder cancers, if attributable to air pollution, is due in particular to traffic, as it is the main source of emission of C_6_H_6_ and NO_2_ [28] while thermal power plant, home heating and diesel-engines are the main sources of emission of SO_2_ [6]. Recent research has shown a possible association between exposure to high level of nitrogen dioxide and excess risk of lung cancer [29]. The contribution of the various sources of pollution is confirmed by the technique of apportionment, i.e., road traffic is responsible for 62% of C_6_H_6_, 55% of NO_2_, and 87% of PM_10_.

This descriptive investigation has some limitations, common to other observational studies. A first important factor not considered in this study is the occupational exposure, and in particular, the exposure to asbestos related to shipbuilding [30], which is associated with increased risks of mesotheliomas and ADK [31,32]. Occupational exposure could affect results differently between males and females.

Secondly, information on smoking, an important confounder, was lacking in this investigation. This variable was, thus, not included in the analysis, because the regional healthcare population database does not report information on this habit. We defined subgroups by sex and age group with different proportions of smokers to minimize smoke confounding. However, this indirect method cannot rule out a tobacco confounder residual effect in the results.

Thirdly, no information was available about the daily time spent in each risk area with different levels of exposures. Finally, the presence of other industries, port, airport and high traffic road near the residential study areas may hamper interpretation of results on the role of air pollution produced by the thermal power plant alone.

An individual human biomonitoring study, with a questionnaire investigating smoking habits, lifestyles, and occupational exposures, is currently underway in the population of the study area. This will allow an evaluation of the actual prevalence of smoking habits in both sexes and classes of age. It is also worth stressing that for lung and bladder cancer cases diagnosed from 1995 to 2009, the relevant period of residential exposure dates at least 30 years before diagnosis (in this case, residential exposures that have occurred between 1965 and 1994). We have therefore assumed that the exposures measured in 1998 were representative of that time period.

However, this study has some strengths. The incidence cases and the study population were derived from two validated regional databases (CR-FVG and healthcare population), which cover overall resident population of the study area. Furthermore, as environmental exposure is concerned, we assumed the residential location of the participants as a proxy of individual exposure. This assumption is based on geocoded residential address and use of mathematical models of air pollution distribution.

## 5. Conclusions

The findings of this descriptive study indicate that air pollution may have a role with regard to the risk of lung and bladder cancers, though limited to women aged ≥75 years. These persons represented approximately 11% of all the cases of lung and bladder cancers in this population. Further analytical investigations are necessary to shed light on the possible determinants of these increased risks, in particular, air pollutants from multiple industrial sources, road, port and airport traffic, home heating, as well as on the role of occupation, smoking habits, and other lifestyles.

## Figures and Tables

**Table 1:** Number of incident cases of lung and bladder carcinomas, age-standardized incidence rates (ASR) with corresponding 95% confidence intervals (CI), by sex and age group. 1995–2009 in 14 municipalities.

	<75 Years						≥75 Years						All Ages					
	Men			Women			Men			Women			Men			Women		
	*N*	ASR	95% CI	*N*	ASR	95% CI	*N*	ASR	95% CI	*N*	ASR	95% CI	*N*	ASR	95% CI	*N*	ASR	95% CI
All cancer *	3813	442.2	(427.9–456.6)	2716	314.9	(302.5–327.4)	1892	3383	(3228.8–3537.3)	1599	1593.4	(1512.4–1674.4)	5705	559.9	(544.8–575)	4315	366.1	(353.7–378.5)
Bladder cancer	304	34.3	(30.4–38.2)	79	8.6	(6.7–10.6)	193	339.5	(291–388)	74	74.2	(56.6–91.8)	497	46.5	(42.3–50.8)	153	11.4	(9.2–13.3)
Lung cancer																		
All lung carcinoma	527	58.4	(53.4–63.5)	153	16	(13.4–18.7)	274	496.5	(437–555.9)	122	126	(102.8–149.1)	801	75.9	(70.5–81.3)	275	20.4	(17.7–23.1)
Adenocarcinoma	131	14.6	(12.1–17.2)	58	6.5	(4.8–8.2)	55	100.7	(73.8–127.6)	24	26.8	(15.7–37.8)	186	18.1	(15.4–20.8)	82	7.3	(5.6–9)
Squamous cell	135	14.7	(12.2–17.2)	23	2.3	(1.3–3.3)	69	129.8	(98.9–160.7)	17	19.9	(10.2–29.6)	204	19.3	(16.6–22)	40	3	(2–4)
Other	261	29	(25.5–32.6)	72	7.2	(5.5–8.9)	150	266	(222.9–309.1)	81	79.3	(61.4–97.2)	411	38.5	(34.7–42.4)	153	10.1	(8.3–11.9)

Note: ASR calculated from age-standardized rates on 2001 EU population; (*): All cancers (non melanoma excluded).

**Table 2:** Number of incident cases of lung cancer, incidence rate ratio (IRR) with corresponding 95% confidence intervals (CI), by sex, tertile of exposure, and age group. 1995–2009 in 14 municipalities. Statistically significant results are reported in bold.

	<75 Years						≥75 Years						All Ages					
	Men			Women			Men			Women			Men			Women		
	*N*	IRR	95% CI	*N*	IRR	95% CI	*N*	IRR	95% CI	*N*	IRR	95% CI	*N*	IRR	95% CI	*N*	IRR	95% CI
**C_6_H_6_ (µg/m^3^)**																		
<1.1	175	1		52	1		97	1		25	1		272	1		77	1	
1.1–1.8	181	1.07	(0.87–1.32)	53	0.94	(0.64–1.38)	89	0.91	(0.71–1.18)	51	**1.86**	(**1.15–3.00**)	270	1.03	(0.87–1.22)	104	1.09	(0.81–1.46)
>1.8	171	1.04	(0.84–1.29)	48	0.95	(0.64–1.41)	88	0.97	(0.75–1.26)	46	**2.00**	(**1.23–3.25**)	259	1.02	(0.86–1.21)	94	1.12	(0.83–1.52)
**NO_2_ (µg/m^3^)**																		
<16.9	172	1		49	1		95	1		28	1		267	1		77	1	
16.9–19.6	184	1.13	(0.92–1.39)	57	1.12	(0.77–1.65)	88	0.93	(0.72–1.20)	50	**1.70**	(**1.07–2.71**)	272	1.07	(0.91–1.27)	107	1.23	(0.92–1.65)
>19.6	171	1.07	(0.86–1.32)	47	0.99	(0.67–1.48)	91	1.04	(0.80–1.34)	44	**1.72**	(**1.07–2.77**)	262	1.06	(0.89–1.25)	91	1.14	(0.84–1.54)
**PM_10_ (µg/m^3^)**																		
<40.6	181	1		58	1		94	1		26	1		275	1		84	1	
40.6–51.9	183	1.03	(0.84–1.27)	51	0.82	(0.57–1.20)	97	1.00	(0.78–1.28)	61	**2.11**	(**1.33–3.33**)	280	1.02	(0.87–1.21)	112	1.02	(0.77–1.36)
>51.9	163	0.99	(0.81–1.23)	44	0.81	(0.55–1.20)	83	1.01	(0.78–1.32)	35	1.57	(0.94–2.60)	246	1.00	(0.84–1.19)	79	0.93	(0.68–1.27)
**SO_2_ (µg/m^3^)**																		
<34.6	184	1		52	1		99	1		27	1		283	1		79	1	
34.6–37.5	175	1.04	(0.85–1.28)	47	0.88	(0.59–1.30)	91	1.01	(0.79–1.30)	45	1.55	(0.96–2.49)	266	1.03	(0.87–1.22)	92	0.98	(0.73–1.33)
>37.5	168	0.97	(0.79–1.20)	54	1.06	(0.72–1.55)	84	0.87	(0.67–1.13)	50	**1.71**	(**1.07–2.73**)	252	0.95	(0.80–1.12)	104	1.16	(0.87–1.56)

**Table 3:** Number of incident cases of bladder cancer incidence rate ratio (IRR) with corresponding 95% confidence intervals (CI), by sex, tertile of exposure, and age group. 1995–2009 in 14 municipalities. Statistically significant results are reported in bold.

	<75 Years						≥75 Years						All Ages					
	Men			Women			Men			Women			Men			Women		
	*N*	IRR	95% CI	*N*	IRR	95% CI	*N*	IRR	95% CI	*N*	IRR	95% CI	*N*	IRR	95% CI	*N*	IRR	95% CI
**C_6_H_6_ (µg/m^3^)**																		
<1.1	102	1		25	1		65	1		14	1		167	1		39	1	
1.1–1.8	110	1.12	(0.86–1.47)	25	0.89	(0.51–1.55)	65	0.96	(0.70–1.31)	35	**2.39**	(**1.29–4.44**)	175	1.07	(0.87–1.33)	60	1.16	(0.77–1.73)
>1.8	92	0.95	(0.71–1.25)	29	1.33	(0.78–2.26)	63	1.05	(0.77–1.44)	25	**1.94**	(**1.01–3.74**)	155	0.98	(0.79–1.22)	54	1.44	(0.95–2.17)
**NO_2_ (µg/m^3^)**																		
<16.9	100	1		25	1		66	1		15	1		166	1		40	1	
16.9–19.6	108	1.13	(0.86–1.49)	22	0.79	(0.44–1.40)	61	0.92	(0.67–1.27)	32	**1.97**	(**1.07–3.63**)	169	1.07	(0.86–1.33)	54	1.02	(0.68–1.54)
>19.6	96	1.02	(0.77–1.35)	32	1.44	(0.85–2.43)	66	1.10	(0.80–1.49)	27	**1.94**	(**1.03–3.65**)	162	1.04	(0.84–1.30)	59	**1.53**	(**1.03–2.29**)
**PM_10_ (µg/m^3^)**																		
<40.6	96	1		27	1		72	1		15	1		168	1		42	1	
40.6–51.9	114	1.18	(0.90–1.55)	27	0.91	(0.53–1.55)	69	0.92	(0.68–1.26)	37	**2.39**	(**1.31–4.35**)	183	1.10	(0.89–1.36)	64	1.16	(0.78–1.71)
>51.9	94	1.06	(0.80–1.41)	25	1.10	(0.64–1.90)	52	0.88	(0.63–1.23)	22	1.78	(0.92–3.44)	146	1.00	(0.80–1.25)	47	1.21	(0.80–1.84)
**SO_2_ (µg/m^3^)**																		
<34.6	98	1		24	1		68	1		18	1		166	1		42	1	
34.6–37.5	115	1.28	(0.98–1.68)	24	1.03	(0.58–1.81)	54	0.89	(0.64–1.24)	32	1.75	(0.98–3.12)	169	1.16	(0.94–1.44)	56	1.19	(0.80–1.78)
>37.5	91	0.97	(0.73–1.29)	31	1.43	(0.84–2.43)	71	1.14	(0.84–1.55)	24	1.27	(0.69–2.34)	162	1.02	(0.82–1.27)	55	1.39	(0.93–2.08)

## Supplementary Materials

The followings are available online at www.mdpi.com/1660-4601/14/8/860/s1: Table S1: Number of incident cases of lung cancer, age standardized rates (ASR) with corresponding 95% confidence intervals (CI), by sex, tertile of exposure and, age group. 1995–2009 in 14 municipalities. Table S2: Number of incident cases of lung carcinomas, age standardized rates (ASR) with corresponding 95% confidence intervals (CI), by sex, morphology, tertile of exposure, and age group. 1995–2009 in 14 municipalities. Table S3: Number of incident cases of lung carcinomas, incidence rate ratio (IRR) with corresponding 95% confidence intervals (CI), by sex, morphology, tertile of exposure, and age group. 1995–2009 in 14 municipalities. Table S4: Number of incident cases of bladder cancer, age standardized rates (ASR) with corresponding 95% confidence intervals (CI), by sex, tertile of exposure, and age group. 1995–2009 in 14 municipalities. Table S5: Annual percent changes (APC) and corresponding 95% confidence intervals (CI), of lung cancer incidence by sex, tertile of exposure, and age group. 1995–2009 in 14 municipalities. Table S6: Annual percent changes (APC) and corresponding 95% confidence intervals (CI), of bladder cancer incidence by sex, tertile of exposure, and age group. 1995–2009 in 14 municipalities.
