# Supplementary material for: Residence in Proximity of a Coal-Oil-Fired Thermal Power Plant and Risk of Lung and Bladder Cancer in North-Eastern Italy. A Population-Based Study: 1995–2009
Source: Int J Environ Res Public Health. 2017 Jul 31;14(8):860. doi: 10.3390/ijerph14080860 (PMC5580564; doi:10.3390/ijerph14080860)
Supplement: Supplementary file 1 [file ijerph-14-00860-s001.zip › Collarile_Suppl_Tabs.docx]

**Table S1**. Number of incident cases of lung cancer, age standardized rates (ASR) with corresponding 95% confidence intervals (CI) by sex, tertile of exposure, and age group. 1995-2009 in 14 municipalities.

|  | < 75 Years | | | | | | >=75 Years | | | | | | All ages | | | | | |
| --- | --- | --- | --- | --- | --- | --- | --- | --- | --- | --- | --- | --- | --- | --- | --- | --- | --- | --- |
|  | Men | | | Women | | | Men | | | Women | | | Men | | | Women | | |
|  | N | ASR | 95% CI | N | ASR | 95% CI | N | ASR | 95% CI | N | ASR | 95% CI | N | ASR | 95% CI | N | ASR | 95% CI |
| **C_6_H_6_ (µg/m3)** |  |  |  |  |  |  |  |  |  |  |  |  |  |  |  |  |  |  |
| <1.1 | 175 | 56.3 | (47.8-64.7) | 52 | 16.6 | (11.9-22.2) | 97 | 517.6 | (412.8-622.4) | 25 | 77.9 | (46.1-109.7) | 272 | 74.7 | (65.6-83.9) | 77 | 19.0 | (14.4-23.7) |
| 1.1-1.8 | 181 | 60.3 | (51.4-69.2) | 53 | 15.6 | (11.2-19.9) | 89 | 473.6 | (374.0-573.1) | 51 | 144.9 | (103.7-186.1) | 270 | 76.8 | (67.4-86.3) | 104 | 20.8 | (16.3-25.2) |
| >1.8 | 171 | 58.7 | (49.8-67.6) | 48 | 15.8 | (11.1-20.4) | 88 | 504.0 | (398.1-607.0) | 46 | 155.5 | (109.3-201.6) | 259 | 76.6 | (67.0-86.1) | 94 | 21.4 | (16.6-26.2) |
| **NO_2_ (µg/m3)** |  |  |  |  |  |  |  |  |  |  |  |  |  |  |  |  |  |  |
| <16.9 | 172 | 54.9 | (46.6-63.2) | 49 | 15.4 | (10.9-19.8) | 95 | 504.6 | (401.5-607.7) | 28 | 85.4 | (52.5-118.2) | 267 | 72.9 | (63.9-81.9) | 77 | 18.2 | (13.7-22.6) |
| 16.9-19.6 | 184 | 61.9 | (52.9-71.0) | 57 | 17.3 | (12.6-21.9) | 88 | 468.3 | (369.3-567.3) | 50 | 145.6 | (103.8-187.4) | 272 | 78.2 | (68.6-87.7) | 107 | 22.4 | (17.6-27.2) |
| >19.6 | 171 | 58.5 | (49.7-67.4) | 47 | 15.3 | (10.8-19.9) | 91 | 522.8 | (414.7-630.9) | 44 | 147.3 | (102.5-192.0) | 262 | 77.1 | (67.6-86.7) | 91 | 20.6 | (15.9-25.3) |
| **PM_10_ (µg/m3)** |  |  |  |  |  |  |  |  |  |  |  |  |  |  |  |  |  |  |
| <40.6 | 181 | 57.9 | (49.4-66.4) | 58 | 18.2 | (13.4-23.1) | 94 | 495.9 | (394.0-597.9) | 26 | 80.2 | (48.2-112.2) | 275 | 75.4 | (66.3-84.6) | 84 | 20.7 | (15.9-25.5) |
| 40.6-51.9 | 183 | 59.9 | (51.1-68.7) | 51 | 15.0 | (10.7-19.4) | 97 | 494.4 | (395.2-593.6) | 61 | 169.0 | (125.2-212.9) | 280 | 77.3 | (67.9-86.6) | 112 | 21.2 | (16.7-25.7) |
| >51.9 | 163 | 57.6 | (48.7-66.5) | 44 | 14.8 | (10.3-19.4) | 83 | 503.3 | (393.9-612.6) | 35 | 125.6 | (82.8-168.5) | 246 | 75.4 | (65.8-85.0) | 79 | 19.3 | (14.6-23.9) |
| **SO_2_  (µg/m3)** |  |  |  |  |  |  |  |  |  |  |  |  |  |  |  |  |  |  |
| <34.6 | 184 | 58.2 | (49.7-66.8) | 52 | 16.4 | (11.8-21.0) | 99 | 517.6 | (414.1-621.1) | 27 | 88.5 | (54.1-122.9) | 283 | 76.6 | (67.4-85.8) | 79 | 19.3 | (14.6-23.9) |
| 34.6-37.5 | 175 | 60.5 | (51.4-69.6) | 47 | 14.4 | (10.1-18.6) | 91 | 524.1 | (415.2-633.0) | 45 | 136.9 | (65.2-178.6) | 266 | 79.0 | (69.3-88.8) | 92 | 19.3 | (14.9-23.7) |
| >37.5 | 168 | 56.7 | (48.0-65.4) | 54 | 17.4 | (12.6-22.2) | 84 | 450.3 | (353.2-547.3) | 50 | 151.3 | (108.1-194.5) | 252 | 72.4 | (63.2-81.6) | 104 | 22.8 | (17.8-27.7) |

Note : ASR calculated on European population 2001

**Table S2**. Number of lung carcinomas, age standardized rates (ASR) with corresponding 95% confidence intervals (CI), by sex, morphology, tertile of exposure, and age group. 1995-2009 in 14 municipalities

|  | < 75 Years | | | | | | >=75 Years | | | | | | All ages | | | | | |
| --- | --- | --- | --- | --- | --- | --- | --- | --- | --- | --- | --- | --- | --- | --- | --- | --- | --- | --- |
|  | Men | | | Women | | | Men | | | Women | | | Men | | | Women | | |
|  | N | ASR | 95% CI | N | ASR | 95% CI | N | ASR | 95% CI | N | ASR | 95% CI | N | ASR | 95% CI | N | ASR | 95% CI |
| **C_6_H_6_ (µg/m3)** |  |  |  |  |  |  |  |  |  |  |  |  |  |  |  |  |  |  |
| Adenocarcinoma |  |  |  |  |  |  |  |  |  |  |  |  |  |  |  |  |  |  |
| <1.1 | 51 | 16.58 | (12-21.2) | 18 | 5.87 | (3.1-8.7) | 16 | 90.49 | (45.7-135.2) | 6 | 22.73 | (4.1-41.3) | 67 | 19.53 | (14.7-24.3) | 24 | 6.54 | (3.8-9.3) |
| 1.1-1.8 | 46 | 15.46 | (10.9-20) | 16 | 5.63 | (2.7-8.5) | 22 | 113.40 | (65.4-161.4) | 9 | 25.99 | (8.4-43.6) | 68 | 19.38 | (14.6-24.1) | 25 | 6.44 | (3.6-9.3) |
| >1.8 | 34 | 11.80 | (7.8-15.8) | 24 | 8.08 | (4.7-11.4) | 17 | 100.70 | (52.6-148.8) | 9 | 32.12 | (10.6-53.6) | 51 | 15.35 | (11-19.7) | 33 | 9.05 | (5.7-12.4) |
| Squamous cell |  |  |  |  |  |  |  |  |  |  |  |  |  |  |  |  |  |  |
| <1.1 | 38 | 12.44 | (8.4-16.5) | 10 | 3.18 | (1.1-5.2) | 24 | 140.73 | (84.1-197.4) | 1 | 4.15 | (0-12.3) | 62 | 17.6 | (13.1-22) | 11 | 3.2 | (1.2-5.2) |
| 1.1-1.8 | 50 | 16.06 | (11.6-20.6) | 9 | 2.45 | (0.8-4.1) | 26 | 142.86 | (87.4-198.3) | 11 | 34.02 | (13.3-54.7) | 76 | 21.1 | (16.3-26) | 20 | 3.7 | (1.9-5.5) |
| >1.8 | 47 | 15.80 | (11.2-20.4) | 4 | 1.29 | (0-2.6) | 19 | 106.80 | (58.5-155.1) | 5 | 20.02 | (2.3-37.7) | 66 | 19.4 | (14.6-24.2) | 9 | 2.0 | (0.6-3.5) |
| Other |  |  |  |  |  |  |  |  |  |  |  |  |  |  |  |  |  |  |
| <1.1 | 86 | 27.26 | (21.4-33.1) | 24 | 7.53 | (4.4-10.7) | 57 | 286.40 | (210.4-362.4) | 18 | 51.06 | (26.6-75.5) | 143 | 37.62 | (31.3-44) | 42 | 9.27 | (6.1-12.4) |
| 1.1-1.8 | 85 | 28.79 | (22.6-35) | 28 | 7.49 | (4.7-10.3) | 41 | 217.31 | (150-284.6) | 31 | 84.90 | (54-115.8) | 126 | 36.33 | (29.8-42.9) | 59 | 10.59 | (7.6-13.6) |
| >1.8 | 90 | 31.15 | (24.6-37.7) | 20 | 6.40 | (3.5-9.3) | 52 | 296.53 | (215.4-377.6) | 32 | 103.31 | (66.5-140.1) | 142 | 41.77 | (34.7-48.8) | 52 | 10.27 | (7.1-13.4) |
| **NO_2_ (µg/m3)** |  |  |  |  |  |  |  |  |  |  |  |  |  |  |  |  |  |  |
| Adenocarcinoma |  |  |  |  |  |  |  |  |  |  |  |  |  |  |  |  |  |  |
| <16.9 | 47 | 14.83 | (10.5-19.1) | 20 | 6.34 | (3.5-9.2) | 16 | 92.20 | (6.1-44.7) | 7 | 25.43 | (46.8-137.6) | 63 | 17.92 | (13.4-22.4) | 27 | 7.11 | (4.2-10) |
| 16.9-19.6 | 48 | 16.62 | (11.8-21.4) | 15 | 5.54 | (2.6-8.4) | 21 | 106.70 | (6.6-40.5) | 8 | 23.55 | (60.5-152.9) | 69 | 20.22 | (15.3-25.2) | 23 | 6.26 | (3.4-9.1) |
| >19.6 | 36 | 12.46 | (8.3-16.6) | 23 | 7.70 | (4.5-10.9) | 18 | 106.54 | (10.6-53.7) | 9 | 32.19 | (57.1-156) | 54 | 16.22 | (11.8-20.6) | 32 | 8.68 | (5.4-11.9) |
| Squamous cell |  |  |  |  |  |  |  |  |  |  |  |  |  |  |  |  |  |  |
| <16.9 | 34 | 11.19 | (7.4-15) | 10 | 3.17 | (1.1-5.2) | 23 | 134.70 | (79.5-189.9) | 1 | 4.12 | (0-12.2) | 57 | 16.13 | (11.8-20.4) | 11 | 3.21 | (1.2-5.2) |
| 16.9-19.6 | 52 | 16.78 | (12.2-21.4) | 8 | 2.26 | (0.7-3.9) | 26 | 143.82 | (88-199.7) | 11 | 34.07 | (13.3-54.8) | 78 | 21.86 | (16.9-26.8) | 19 | 3.53 | (1.8-5.3) |
| >19.6 | 49 | 16.43 | (11.8-21.1) | 5 | 1.52 | (0.1-2.9) | 20 | 112.98 | (63.1-162.8) | 5 | 20.06 | (2.3-37.8) | 69 | 20.29 | (15.4-25.2) | 10 | 2.26 | (0.8-3.8) |
| Other |  |  |  |  |  |  |  |  |  |  |  |  |  |  |  |  |  |  |
| <16.9 | 91 | 28.89 | (22.9-34.9) | 19 | 5.85 | (3.1-8.6) | 56 | 277.71 | (203.4-352) | 20 | 55.80 | (30.4-81.2) | 147 | 38.84 | (32.3-45.3) | 39 | 7.85 | (5-10.7) |
| 16.9-19.6 | 84 | 28.53 | (22.4-34.7) | 34 | 9.47 | (6.2-12.8) | 41 | 217.75 | (150.3-285.2) | 31 | 88.01 | (55.9-120.1) | 125 | 36.10 | (29.6-42.6) | 65 | 12.61 | (9.2-16) |
| >19.6 | 86 | 29.66 | (23.3-36) | 19 | 6.13 | (3.3-9) | 53 | 303.31 | (221.1-385.5) | 30 | 95.01 | (60.1-130) | 139 | 40.61 | (33.7-47.5) | 49 | 9.68 | (6.6-12.8) |
| **PM_10_ (µg/m3)** |  |  |  |  |  |  |  |  |  |  |  |  |  |  |  |  |  |  |
| Adenocarcinoma |  |  |  |  |  |  |  |  |  |  |  |  |  |  |  |  |  |  |
| <40.6 | 49 | 15.65 | (11.2-20.1) | 21 | 6.89 | (3.9-9.9) | 18 | 99.26 | (53-145.5) | 6 | 22.06 | (4-40.1) | 67 | 18.99 | (14.4-23.6) | 27 | 7.50 | (4.5-10.5) |
| 40.6-51.9 | 48 | 15.96 | (11.4-20.6) | 17 | 5.66 | (2.9-8.4) | 22 | 114.01 | (66-162) | 13 | 38.56 | (17-60.1) | 70 | 19.88 | (15.1-24.7) | 30 | 6.98 | (4.2-9.8) |
| >51.9 | 34 | 12.17 | (8-16.3) | 20 | 7.05 | (3.8-10.3) | 15 | 89.04 | (43.4-134.7) | 5 | 17.51 | (1.6-33.4) | 49 | 15.24 | (10.9-19.6) | 25 | 7.47 | (4.3-10.6) |
| Squamous cell |  |  |  |  |  |  |  |  |  |  |  |  |  |  |  |  |  |  |
| <40.6 | 45 | 14.64 | (10.3-19) | 11 | 3.32 | (1.3-5.4) | 25 | 143.84 | (87.1-200.5) | 2 | 8.05 | (0-19.2) | 70 | 19.81 | (15.1-24.6) | 13 | 3.51 | (1.5-5.5) |
| 40.6-51.9 | 45 | 14.01 | (9.9-18.1) | 6 | 1.79 | (0.3-3.3) | 19 | 96.87 | (53-140.8) | 13 | 40.20 | (17.8-62.6) | 64 | 17.32 | (13-21.7) | 19 | 3.33 | (1.6-5) |
| >51.9 | 45 | 15.66 | (11-20.3) | 6 | 1.89 | (0.3-3.4) | 25 | 155.52 | (94.1-217) | 2 | 7.79 | (0-18.8) | 70 | 21.26 | (16.2-26.3) | 8 | 2.12 | (0.6-3.7) |
| Other |  |  |  |  |  |  |  |  |  |  |  |  |  |  |  |  |  |  |
| <40.6 | 87 | 27.61 | (21.7-33.5) | 26 | 8.01 | (4.8-11.2) | 51 | 252.85 | (181.9-323.8) | 18 | 50.09 | (26.2-74) | 138 | 36.62 | (30.3-42.9) | 44 | 9.69 | (6.5-12.9) |
| 40.6-51.9 | 90 | 29.93 | (23.6-36.2) | 28 | 7.58 | (4.6-10.5) | 56 | 283.49 | (208.6-358.4) | 35 | 90.25 | (59.3-121.2) | 146 | 40.07 | (33.3-46.8) | 63 | 10.89 | (7.8-14) |
| >51.9 | 84 | 29.76 | (23.3-36.2) | 18 | 5.89 | (3.1-8.7) | 43 | 258.69 | (180.7-336.7) | 28 | 100.34 | (62.1-138.6) | 127 | 38.92 | (32-45.8) | 46 | 9.67 | (6.6-12.8) |
| **SO_2_  (µg/m3)** |  |  |  |  |  |  |  |  |  |  |  |  |  |  |  |  |  |  |
| Adenocarcinoma |  |  |  |  |  |  |  |  |  |  |  |  |  |  |  |  |  |  |
| <34.6 | 52 | 16.38 | (11.9-20.9) | 20 | 6.46 | (3.5-9.4) | 18 | 93.31 | (49.5-137.1) | 6 | 22.59 | (4.2-41) | 70 | 19.46 | (14.8-24.1) | 26 | 7.10 | (4.2-10) |
| 34.6-37.5 | 49 | 17.24 | (12.4-22.1) | 15 | 4.92 | (2.3-7.5) | 16 | 94.80 | (47.9-141.7) | 8 | 23.43 | (6.4-40.5) | 65 | 20.34 | (15.3-25.4) | 23 | 5.66 | (3.1-8.2) |
| >37.5 | 30 | 10.29 | (6.6-14) | 23 | 8.15 | (4.7-11.6) | 21 | 115.02 | (65.4-164.6) | 10 | 33.78 | (12.4-55.2) | 51 | 14.48 | (10.4-18.6) | 33 | 9.17 | (5.8-12.6) |
| Squamous cell |  |  |  |  |  |  |  |  |  |  |  |  |  |  |  |  |  |  |
| <34.6 | 43 | 13.80 | (9.6-18) | 9 | 2.86 | (0.9-4.8) | 24 | 136.19 | (81.4-191) | 6 | 23.32 | (4.5-42.1) | 67 | 18.70 | (14.1-23.3) | 15 | 3.68 | (1.6-5.7) |
| 34.6-37.5 | 41 | 13.58 | (9.4-17.8) | 8 | 2.30 | (0.7-3.9) | 25 | 144.81 | (87.4-202.2) | 8 | 27.46 | (7.8-47.1) | 66 | 18.83 | (14.2-23.4) | 16 | 3.31 | (1.6-5.1) |
| >37.5 | 51 | 16.93 | (12.2-21.7) | 6 | 1.82 | (0.3-3.3) | 20 | 109.70 | (61.3-158.1) | 3 | 9.24 | (0-19.9) | 71 | 20.64 | (15.7-25.6) | 9 | 2.12 | (0.6-3.6) |
| Other |  |  |  |  |  |  |  |  |  |  |  |  |  |  |  |  |  |  |
| <34.6 | 89 | 28.06 | (22.2-34) | 23 | 7.08 | (4.1-10.1) | 57 | 288.12 | (211.9-364.3) | 15 | 42.59 | (20.4-64.8) | 146 | 38.46 | (32-44.9) | 38 | 8.50 | (5.5-11.5) |
| 34.6-37.5 | 85 | 29.68 | (23.3-36.1) | 24 | 7.13 | (4.2-10.1) | 50 | 284.48 | (204.8-364.2) | 29 | 86.02 | (53.4-118.6) | 135 | 39.87 | (33-46.8) | 53 | 10.29 | (7.2-13.4) |
| >37.5 | 87 | 29.45 | (23.2-35.7) | 25 | 7.43 | (4.4-10.5) | 43 | 225.55 | (157.6-293.5) | 37 | 108.29 | (72.3-144.3) | 130 | 37.29 | (30.7-43.9) | 62 | 11.46 | (8.2-14.7) |

**Table S3**. Number of Incident cases of lung carcinomas, incidence rate ratio (IRR) with corresponding 95% confidence intervals (CI), by sex, morphology, tertile of exposure, and age group. 1995-2009 in 14 municipalities. Statistically significant results are reported in bold.

|  | < 75 Years | | | | | | >=75 Years | | | | | | All ages | | | | | |
| --- | --- | --- | --- | --- | --- | --- | --- | --- | --- | --- | --- | --- | --- | --- | --- | --- | --- | --- |
|  | Men | | | Women | | | Men | | | Women | | | Men | | | Women | | |
|  | N | IRR | 95% CI | N | IRR | 95% CI | N | IRR | 95% CI | N | IRR | 95% CI | N | IRR | 95% CI | N | IRR | 95% CI |
| **C_6_H_6_ (µg/m3)** |  |  |  |  |  |  |  |  |  |  |  |  |  |  |  |  |  |  |
| Adenocarcinoma |  |  |  |  |  |  |  |  |  |  |  |  |  |  |  |  |  |  |
| <1.1 | 51 | 1 |  | 18 | 1 |  | 16 | 1 |  | 6 | 1 |  | 67 | 1 |  | 24 | 1 |  |
| 1.1-1.8 | 46 | 0.93 | (0.63-1.39) | 16 | 0.96 | (0.49-1.88) | 22 | 1.25 | (0.66-2.39) | 9 | 1.14 | (0.41-3.21) | 68 | 0.99 | (0.71-1.39) | 25 | 0.99 | (0.56-1.73) |
| >1.8 | 34 | 0.71 | (0.46-1.1) | 24 | 1.38 | (0.75-2.54) | 17 | 1.11 | (0.56-2.2) | 9 | 1.24 | (0.44-3.47) | 51 | 0.79 | (0.55-1.13) | 33 | 1.38 | (0.82-2.34) |
| Squamous cell |  |  |  |  |  |  |  |  |  |  |  |  |  |  |  |  |  |  |
| <1.1 | 38 | 1 |  | 10 | 1 |  | 24 | 1 |  | 1 | 1 |  | 62 | 1 |  | 11 | 1 |  |
| 1.1-1.8 | 50 | 1.29 | (0.85-1.97) | 9 | 0.77 | (0.31-1.9) | 26 | 1.02 | (0.58-1.77) | 11 | **8.20** | **(1.06-63.52)** | 76 | 1.20 | (0.86-1.68) | 20 | 1.16 | (0.55-2.41) |
| >1.8 | 47 | 1.27 | (0.83-1.95) | 4 | 0.41 | (0.13-1.29) | 19 | 0.76 | (0.42-1.39) | 5 | 4.83 | (0.56-41.31) | 66 | 1.11 | (0.78-1.56) | 9 | 0.63 | (0.26-1.53) |
| Other |  |  |  |  |  |  |  |  |  |  |  |  |  |  |  |  |  |  |
| <1.1 | 86 | 1 |  | 24 | 1 |  | 57 | 1 |  | 18 | 1 |  | 143 | 1 |  | 42 | 1 |  |
| 1.1-1.8 | 85 | 1.06 | (0.78-1.43) | 28 | 1.00 | (0.58-1.72) | 41 | 0.76 | (0.51-1.13) | 31 | 1.66 | (0.93-2.97) | 126 | 0.97 | (0.76-1.23) | 59 | 1.14 | (0.77-1.7) |
| >1.8 | 90 | 1.14 | (0.85-1.54) | 20 | 0.85 | (0.47-1.54) | 52 | 1.04 | (0.71-1.51) | 32 | **2.02** | **(1.14-3.6)** | 142 | 1.11 | (0.88-1.4) | 52 | 1.11 | (0.74-1.66) |
| **NO_2_ (µg/m3)** |  |  |  |  |  |  |  |  |  |  |  |  |  |  |  |  |  |  |
| Adenocarcinoma |  |  |  |  |  |  |  |  |  |  |  |  |  |  |  |  |  |  |
| <16.9 | 47 | 1 |  | 20 | 1 |  | 16 | 1 |  | 7 | 1 |  | 63 | 1 |  | 27 | 1 |  |
| 16.9-19.6 | 48 | 1.12 | (0.75-1.68) | 15 | 0.87 | (0.45-1.71) | 21 | 1.16 | (0.6-2.22) | 8 | 0.93 | (0.34-2.55) | 69 | 1.13 | (0.8-1.59) | 23 | 0.88 | (0.51-1.54) |
| >19.6 | 36 | 0.84 | (0.54-1.3) | 23 | 1.21 | (0.67-2.21) | 18 | 1.16 | (0.59-2.27) | 9 | 1.37 | (0.51-3.67) | 54 | 0.91 | (0.63-1.3) | 32 | 1.22 | (0.73-2.04) |
| Squamous cell |  |  |  |  |  |  |  |  |  |  |  |  |  |  |  |  |  |  |
| <16.9 | 34 | 1 |  | 10 | 1 |  | 23 | 1 |  | 1 | 1 |  | 57 | 1 |  | 11 | 1 |  |
| 16.9-19.6 | 52 | 1.50 | (0.97-2.31) | 8 | 0.71 | (0.28-1.81) | 26 | 1.07 | (0.61-1.87) | 11 | **8.26** | **(1.07-63.99)** | 78 | 1.36 | (0.96-1.91) | 19 | 1.10 | (0.52-2.31) |
| >19.6 | 49 | 1.47 | (0.95-2.27) | 5 | 0.48 | (0.16-1.4) | 20 | 0.84 | (0.46-1.53) | 5 | 4.86 | (0.57-41.63) | 69 | 1.26 | (0.89-1.79) | 10 | 0.71 | (0.3-1.66) |
| Other |  |  |  |  |  |  |  |  |  |  |  |  |  |  |  |  |  |  |
| <16.9 | 91 | 1 |  | 19 | 1 |  | 56 | 1 |  | 20 | 1 |  | 147 | 1 |  | 39 | 1 |  |
| 16.9-19.6 | 84 | 0.99 | (0.73-1.33) | 34 | 1.62 | (0.92-2.84) | 41 | 0.78 | (0.52-1.17) | 31 | 1.58 | (0.9-2.77) | 125 | 0.93 | (0.73-1.18) | 65 | **1.61** | **(1.08-2.39)** |
| >19.6 | 86 | 1.03 | (0.76-1.38) | 19 | 1.05 | (0.55-1.98) | 53 | 1.09 | (0.75-1.59) | 30 | 1.70 | (0.97-3) | 139 | 1.05 | (0.83-1.32) | 49 | 1.23 | (0.81-1.88) |
| **PM_10_ (µg/m3)** |  |  |  |  |  |  |  |  |  |  |  |  |  |  |  |  |  |  |
| Adenocarcinoma |  |  |  |  |  |  |  |  |  |  |  |  |  |  |  |  |  |  |
| <40.6 | 49 | 1 |  | 21 | 1 |  | 18 | 1 |  | 6 | 1 |  | 67 | 1 |  | 27 | 1 |  |
| 40.6-51.9 | 48 | 1.02 | (0.68-1.52) | 17 | 0.82 | (0.43-1.56) | 22 | 1.15 | (0.62-2.14) | 13 | 0.57 | (0.66-4.6) | 70 | 1.05 | (0.75-1.46) | 30 | 0.93 | (0.55-1.56) |
| >51.9 | 34 | 0.78 | (0.5-1.2) | 20 | 1.02 | (0.55-1.89) | 15 | 0.90 | (0.45-1.78) | 5 | 2.20 | (0.24-2.6) | 49 | 0.80 | (0.56-1.16) | 25 | 1.00 | (0.58-1.72) |
| Squamous cell |  |  |  |  |  |  |  |  |  |  |  |  |  |  |  |  |  |  |
| <40.6 | 45 | 1 |  | 11 | 1 |  | 25 | 1 |  | 2 | 1 |  | 70 | 1 |  | 13 | 1 |  |
| 40.6-51.9 | 45 | 0.96 | (0.63-1.45) | 6 | 0.54 | (0.2-1.46) | 19 | 0.67 | (0.37-1.22) | 13 | **4.99** | **(1.13-22.13)** | 64 | 0.87 | (0.62-1.23) | 19 | 0.95 | (0.47-1.92) |
| >51.9 | 45 | 1.07 | (0.71-1.62) | 6 | 0.57 | (0.21-1.54) | 25 | 1.08 | (0.62-1.88) | 2 | 0.97 | (0.14-6.87) | 70 | 1.07 | (0.77-1.49) | 8 | 0.61 | (0.25-1.46) |
| Other |  |  |  |  |  |  |  |  |  |  |  |  |  |  |  |  |  |  |
| <40.6 | 87 | 1 |  | 26 | 1 |  | 51 | 1 |  | 18 | 1 |  | 138 | 1 |  | 44 | 1 |  |
| 40.6-51.9 | 90 | 1.08 | (0.81-1.46) | 28 | 0.95 | (0.56-1.61) | 56 | 1.12 | (0.77-1.64) | 35 | **1.80** | **(1.02-3.18)** | 146 | 1.09 | (0.87-1.38) | 63 | 1.12 | (0.76-1.65) |
| >51.9 | 84 | 1.08 | (0.8-1.45) | 18 | 0.74 | (0.4-1.34) | 43 | 1.02 | (0.68-1.54) | 28 | **2.00** | **(1.11-3.62)** | 127 | 1.06 | (0.84-1.35) | 46 | 1.00 | (0.66-1.51) |
| **SO_2_  (µg/m3)** |  |  |  |  |  |  |  |  |  |  |  |  |  |  |  |  |  |  |
| Adenocarcinoma |  |  |  |  |  |  |  |  |  |  |  |  |  |  |  |  |  |  |
| <34.6 | 52 | 1 |  | 20 | 1 |  | 18 | 1 |  | 6 | 1 |  | 70 | 1 |  | 26 | 1 |  |
| 34.6-37.5 | 49 | 1.05 | (0.71-1.56) | 15 | 0.76 | (0.39-1.49) | 16 | 1.02 | (0.52-1.99) | 8 | 1.04 | (0.36-2.99) | 65 | 1.05 | (0.75-1.47) | 23 | 0.80 | (0.45-1.4) |
| >37.5 | 30 | 0.63 | (0.4-0.99) | 23 | 1.26 | (0.69-2.3) | 21 | 1.23 | (0.66-2.31) | 10 | 1.44 | (0.54-4.11) | 51 | 0.74 | (0.52-1.07) | 33 | 1.29 | (0.77-2.16) |
| Squamous cell |  |  |  |  |  |  |  |  |  |  |  |  |  |  |  |  |  |  |
| <34.6 | 43 | 1 |  | 9 | 1 |  | 24 | 1 |  | 6 | 1 |  | 67 | 1 |  | 15 | 1 |  |
| 34.6-37.5 | 41 | 0.98 | (0.64-1.51) | 8 | 0.81 | (0.31-2.09) | 25 | 1.06 | (0.61-1.86) | 8 | 1.18 | (0.41-3.39) | 66 | 1.01 | (0.72-1.41) | 16 | 0.90 | (0.45-1.82) |
| >37.5 | 51 | 1.23 | (0.82-1.84) | 6 | 0.64 | (0.23-1.79) | 20 | 0.81 | (0.45-1.46) | 3 | 0.40 | (0.1-1.58) | 71 | 1.10 | (0.79-1.54) | 9 | 0.58 | (0.25-1.32) |
| Other |  |  |  |  |  |  |  |  |  |  |  |  |  |  |  |  |  |  |
| <34.6 | 89 | 1 |  | 23 | 1 |  | 57 | 1 |  | 15 | 1 |  | 146 | 1 |  | 38 | 1 |  |
| 34.6-37.5 | 85 | 1.06 | (0.79-1.42) | 24 | 1.01 | (0.57-1.78) | 50 | 0.99 | (0.68-1.44) | 29 | **2.02** | **(1.08-3.77)** | 135 | 1.04 | (0.82-1.31) | 53 | 1.21 | (0.8-1.84) |
| >37.5 | 87 | 1.05 | (0.78-1.41) | 25 | 1.05 | (0.6-1.85) | 43 | 0.78 | (0.53-1.16) | 37 | **2.54** | **(1.4-4.63)** | 130 | 0.97 | (0.77-1.23) | 62 | 1.35 | (0.9-2.02) |

**Table S4**. Number of incident cases of bladder cancer, age standardized rates (ASR) with corresponding 95% confidence intervals (CI) by sex, tertile of exposure, and age group. 1995-2009 in 14 municipalities

|  | < 75 Years | | | | | | >=75 Years | | | | | | All ages | | | | | |
| --- | --- | --- | --- | --- | --- | --- | --- | --- | --- | --- | --- | --- | --- | --- | --- | --- | --- | --- |
|  | Men | | | Women | | | Men | | | Women | | | Men | | | Women | | |
|  | N | ASR | 95% CI | N | ASR | 95% CI | N | ASR | 95% CI | N | ASR | 95% CI | N | ASR | 95% CI | N | ASR | 95% CI |
| **C_6_H_6_ (µg/m3)** |  |  |  |  |  |  |  |  |  |  |  |  |  |  |  |  |  |  |
| <1.1 | 102 | 33.5 | (26.9-40.1) | 25 | 8.0 | (4.8-11.2) | 65 | 338.9 | (254.7-423.1) | 14 | 41.6 | (18.9-64.0) | 167 | 45.7 | (38.5-52.9) | 39 | 9.3 | (6.1-12.6) |
| 1.1-1.8 | 110 | 37.6 | (30.5-44.7) | 25 | 7.1 | (4.2-10.0) | 65 | 324.9 | (244.9-405.0) | 35 | 99.4 | (65.3-133.6) | 175 | 49.1 | (41.5-56.6) | 60 | 10.8 | (7.7-13.9) |
| >1.8 | 92 | 31.7 | (25.2-38.3) | 29 | 10.6 | (6.6-14.6) | 63 | 356.3 | (267.8-444.8) | 25 | 80.8 | (48.1-113.6) | 155 | 44.7 | (37.5-52.0) | 54 | 13.4 | (9.4-17.5) |
| **NO_2_ (µg/m3)** |  |  |  |  |  |  |  |  |  |  |  |  |  |  |  |  |  |  |
| <16.9 | 100 | 32.6 | (26.1-39.1) | 25 | 8.0 | (4.7-11.2) | 66 | 338.2 | (254.8-421.6) | 15 | 45.5 | (21.6-69.4) | 166 | 44.9 | (37.8-51.9) | 40 | 9.5 | (6.2-12.7) |
| 16.9-19.6 | 108 | 37.0 | (29.9-44.1) | 22 | 6.3 | (3.6-9.1) | 61 | 312.6 | (233.2-392.1) | 32 | 89.5 | (57.3-121.7) | 169 | 48.1 | (40.6-55.6) | 54 | 9.7 | (6.8-12.6) |
| >19.6 | 96 | 33.3 | (26.5-40.0) | 32 | 11.5 | (7.3-15.6) | 66 | 370.5 | (280.5-460.4) | 27 | 88.3 | (53.9-122.7) | 162 | 46.8 | (39.4-54.2) | 59 | 14.5 | (10.3-18.7) |
| **PM_10_ (µg/m3)** |  |  |  |  |  |  |  |  |  |  |  |  |  |  |  |  |  |  |
| <40.6 | 96 | 31.7 | (25.3-38.2) | 27 | 8.6 | (5.3-12.0) | 72 | 363.7 | (278.0-449.5) | 15 | 42.8 | (20.3-65.2) | 168 | 45.0 | (37.9-52.1) | 42 | 10.0 | (6.6-13.4) |
| 40.6-51.9 | 114 | 37.5 | (30.5-44.6) | 27 | 7.8 | (4.7-10.9) | 69 | 336.1 | (256.1-415.0) | 37 | 102.1 | (68.0-136.3) | 183 | 49.5 | (42.0-56.9) | 64 | 11.6 | (8.3-14.8) |
| >51.9 | 94 | 33.6 | (26.8-40.5) | 25 | 9.5 | (5.6-13-3) | 52 | 319.0 | (231.5-406.4) | 22 | 76.3 | (43.4-109.3) | 146 | 45.0 | (37.6-52.5) | 47 | 12.1 | (8.2-16.0) |
| **SO_2_  (µg/m3)** |  |  |  |  |  |  |  |  |  |  |  |  |  |  |  |  |  |  |
| <34.6 | 98 | 31.7 | (25.4-38.1) | 24 | 7.5 | (4.4-10.6) | 68 | 335.2 | (253.9-416.5) | 18 | 55.4 | (28.8-82.0) | 166 | 43.9 | (37.0-50.8) | 42 | 9.4 | (6.2-12.6) |
| 34.6-37.5 | 115 | 40.7 | (33.1-48.2) | 24 | 7.7 | (4.5-10.8) | 54 | 298.6 | (218.1-379.2) | 32 | 97.0 | (62.0-131.9) | 169 | 51.0 | (43.1-58.9) | 56 | 11.2 | (7.9-14.6) |
| >37.5 | 91 | 30.8 | (24.4-37.2) | 31 | 10.7 | (6.7-14.7) | 71 | 382.9 | (293.1-472.7) | 24 | 70.4 | (41.2-100.0) | 162 | 44.9 | (37.8-52.0) | 55 | 13.1 | (9.1-17.1) |

Note : ASR calculated on European population 2001

**Table S5**. Annual percent changes (APC) and corresponding 95% confidence intervals (CI) of lung cancer incidence, by sex, tertile of exposure, and age group. 1995-2009 in 14 municipalities. Statistically significant results are reported in bold.

|  | <75 Years | | | | >= 75 Years | | | | All ages | | | |
| --- | --- | --- | --- | --- | --- | --- | --- | --- | --- | --- | --- | --- |
|  | Men | | Women | | Men | | Women | | Men | | Women | |
|  | APC | 95% CI | APC | 95% CI | APC | 95% CI | APC | 95% CI | APC | 95% CI | APC | 95% CI |
| **C_6_H_6_ (µg/m3)** |  |  |  |  |  |  |  |  |  |  |  |  |
| <1.1 | **-5.29** | (**-9.3; -1.1**) | **-4.77** | (**-9.1; -0.2**) | +0.14 | (-4.6 ; +5.1) | -3.19 | (-10.6; +4.9) | -4.22 | (-7.3 ; -1.1) | -5.08 | (-8.7 ; -1.4) |
| 1.1-1.8 | **-5.29** | (**-9.2; -1.2**) | +0.41 | (-5.8; +7.0) | +3.77 | (-0.9 ; +8.6) | +0.76 | (-6.5 ; +8.5) | -2.98 | (-6.7 ; +0.9) | -0.67 | (-6.4 ; +5.4) |
| >1.8 | -2.13 | (-5.3; +1.1) | -1.76 | (-6.5; +3.2) | -3.74 | (-9.7 ; +2.6) | -1.36 | (-6.6 ; +4.2) | -2.27 | (-5.1 ; +0.6) | -1.82 | (-6.0 ; +2.5) |
| **NO_2_ (µg/m3)** |  |  |  |  |  |  |  |  |  |  |  |  |
| <17.0 | **-5.56** | (**-9.2 ; -1.7**) | -4.55 | (-9.5 ; +0.7) | +1.06 | (-3.4 ; +5.7) | -6.02 | (-13.5; +2.1) | -3.99 | (-6.7 ; -1.2) | -5.54 | (-9.9 ; -1.0) |
| 17.0-19.6 | **-5.50** | (**-9.6 ; -1.2**) | +0.43 | (-5.9 ; +7.2) | -0.37 | (-5.7 ; +5.3) | +2.65 | (-4.1 ; +9.9) | -4.22 | (-7.9 ; -0.4) | -0.83 | (-5.8 ; +4.4) |
| >19.6 | -1.87 | (-5.1 ; +1.5) | -1.61 | (-7.0 ; +4.0) | -1.31 | (-6.9 ; +4.6) | -1.33 | (-7.2 ; +4.9) | -1.44 | (-4.6 ; +1.8) | -1.72 | (-5.9 ; +2.6) |
| **PM_10_ (µg/m3)** |  |  |  |  |  |  |  |  |  |  |  |  |
| <40.6 | **-7.07** | (**-10.9 ; -3.1**) | -4.17 | (-8.7 ; +0.6) | +1.52 | (-3.2 ; +6.4) | -3.10 | (-10.7; +5.1) | -5.22 | (-8.1 ; -2.2) | -4.10 | (-8.4 ; +0.4) |
| 40.6-51.9 | -2.85 | (-7.4 ; +1.9) | +0.40 | (-5.9 ; +7.1) | +1.22 | (-3.1 ; +5.8) | -1.05 | (-5.0 ; +3.1) | -2.04 | (-6.0 ; -2.1) | -1.44 | (-6.5 ; +3.9) |
| >51.9 | -2.68 | (-5.5 ; +0.3) | -2.30 | (-8.6 ; +4.4) | -1.42 | (-6.2 ;+3.7) | -1.55 | (-9.8 ; +7.4) | -2.23 | (-4.6 ; +0.2) | -2.54 | (-8.3 ; +3.6) |
| **SO_2_  (µg/m3)** |  |  |  |  |  |  |  |  |  |  |  |  |
| <34.6 | **-5.40** | (**-9.3 ; -1.4**) | **-8.49** | (**-12.6 ; -4.2**) | -0.43 | (-4.8 ; +4.2) | -2.22 | (-8.4 ; +4.4) | -4.25 | (-7.4 ; -1.0) | -7.60 | (-12.5 ; -2.4) |
| 34.6-37.5 | **-4.80** | (**-7.0 ; -2.6**) | +5.07 | (-1.5; +12.1) | +0.24 | (-4.9 ; +5.7) | -3.84 | (-9.9 ; +2.7) | -3.47 | (-6.0 ; -0.9) | +2.17 | (-2.0 ; +6.5) |
| >37.5 | -3.83 | (-7.8 ; +0.3) | -1.99 | (-10.0; +6.8) | +1.76 | (-3.5 ; +7.3) | +1.99 | (-5.0 ; +9.5) | -2.48 | (-5.2 ; 0.3) | -0.45 | (-6.2 ; +5.7) |

**Table S6**. Annual percent changes (APC) and corresponding 95% confidence intervals (CI) of bladder cancer incidence, by sex, tertile of exposure, and age group. 1995-2009 in 14 municipalities. Statistically significant results are reported in bold.

|  | <75 Years | | | | >= 75 Years | | | | All ages | | | |
| --- | --- | --- | --- | --- | --- | --- | --- | --- | --- | --- | --- | --- |
|  | Men | | Women | | Men | | Women | | Men | | Women | |
|  | APC | 95% CI | APC | 95% CI | APC | 95% CI | APC | 95% CI | APC | 95% CI | APC | 95% CI |
| **C_6_H_6_ (µg/m3)** |  |  |  |  |  |  |  |  |  |  |  |  |
| <1.1 | -0.81 | (-6.7 ; +5.5) | +2.17 | (-5.3 ; +10.3) | -1.27 | (-7.9 ; +5.9) | +0.05 | (-10.7; +12.1) | -0.32 | (-4.0; +3.5) | +2.81 | (-4.6; +10.8) |
| 1.1-1.8 | **-5.07** | (**-9.7 ; -0.2**) | +1.05 | (-9.8 ; +13.2) | -1.91 | (-9.4 ; +6.2) | +1.85 | (-5.8 ; +10.1) | -3.70 | (-7.8; +0.6) | +2.64 | (-6.6; +12.8) |
| >1.8 | -1.31 | (-5.5 ; +3.1) | +2.30 | (-3.3 ; +8.2) | +0.41 | (-5.2 ; +6.4) | -5.53 | (-13.7 ; +3.4) | -0.75 | (-4.7; +3.4) | -0.88 | (-7.8; +6.6) |
| **NO_2_ (µg/m3)** |  |  |  |  |  |  |  |  |  |  |  |  |
| <17.0 | -1.36 | (-6.5 ; +4.1) | +0.61 | (-5.9 ; +7.6) | -1.34 | (-7.9 ; +5.7) | -2.16 | (-15.8 ; +13.7) | -0.90 | (-4.3; +2.7) | +1.45 | (-5.2; +8.6) |
| 17.0-19.6 | **-4.55** | (**-8.6 ; -0.3**) | +1.62 | (-7.8 ; +12.0) | -2.40 | (-9.8 ; +5.7) | +2.27 | (-6.5 ; +11.8) | -3.47 | (-6.9; +0.1) | +3.05 | (-4.4; +11.1) |
| >19.6 | -0.63 | (-4.7 ; +3.6) | +4.05 | (-2.4 ; +10.9) | +0.88 | (-4.5 ; +6.6) | -6.92 | (-14.3 ; +1.1) | -0.19 | (-4.0; +3.8) | -0.92 | (-7.4; +6.0) |
| **PM_10_ (µg/m3)** |  |  |  |  |  |  |  |  |  |  |  |  |
| <40.6 | -1.05 | (-7.3 ; +5.6) | +1.70 | (-6.0 ; +10.0) | -2.89 | (-7.4 ; +1.8) | +1.73 | (-7.0 ; +11.3) | -0.88 | (-4.7; +3.1) | +1.81 | (-5.9; +10.2) |
| 40.6-51.9 | -2.63 | (-6.6 ; +1.5) | +2.35 | (-6.2 ; +11.6) | -1.57 | (-8.4 ; +5.8) | -6.30 | (-12.7 ; +0.6) | -1.79 | (-5.0; +1.6) | -0.20 | (-6.6; +6.6) |
| >51.9 | -3.60 | (-8.6 ; +1.7) | -0.51 | (-6.9 ; +6.3) | +4.11 | (-1.5; +10.1) | +6.47 | (-0.4 ; +13.9) | -1.64 | (-6.4; +3.4) | +0.84 | (-5.9; +8.0) |
| **SO_2_  (µg/m3)** |  |  |  |  |  |  |  |  |  |  |  |  |
| <34.6 | +0.61 | (-5.5 ; +7.1) | +0.70 | (-4.7 ; +6.5) | -1.73 | (-7.4 ; +4.3) | +4.45 | (-3.5 ; +13.1) | +0.31 | (-4.3; +5.1) | +3.79 | (-3.8; +12.0) |
| 34.6-37.5 | -3.51 | (-8.0 ; +1.1) | +5.02 | (-5.0 ; +16.1) | -3.00 | (-9.6 ; +4.1) | +0.08 | (-7.3 ; +9.8) | -3.36 | (-7.5; +0.9) | +6.63 | (-0.7; +14.5) |
| >37.5 | -3.37 | (-8.4 ; +2.0) | -4.40 | (-11.3 ; +3.1) | +3.13 | (-1.1 ; +7.6) | **-9.96** | (**-16.9 ; -2.4**) | -0.99 | (-5.4; +3.7) | -4.47 | (-11.0; +2.5) |
